# The Influence of Lipid Electric Charge on the Binding of Aβ(1–42) Amyloid Peptide to Bilayers in the Liquid-Ordered State

**DOI:** 10.3390/biom14030298

**Published:** 2024-03-01

**Authors:** Hasna Ahyayauch, Massimo E. Masserini, Félix M. Goñi, Alicia Alonso

**Affiliations:** 1 Departamento de Bioquímica, Instituto Biofisika (CSIC, UPV/EHU), Universidad del País Vasco, 48940 Leioa, Spain; ahyayauch@hotmail.com (H.A.); alicia.alonso@ehu.eus (A.A.); 2 Institut Supérieur des Professions Infirmières et Techniques de Santé, Rabat 60000, Morocco; 3 Laboratoire de Biologie et Santé, Unité Neurosciences, Neuroimmunologie et Comportement, Faculty of Sciences, Ibn Tofail University, Kénitra 14000, Morocco; 4 School of Medicine and Surgery, University of Milano-Bicocca, 20900 Monza, Italy; massimo.masserini@unimib.it

**Keywords:** Aβ42, β-amyloid, Aβ membrane binding, ganglioside, sphingomyelin, cholesterol, isothermal calorimetry, Langmuir balance, Alzheimer’s disease

## Abstract

The amyloidogenic Aβ peptides are widely considered as a pathogenic agent in Alzheimer’s disease. Aβ(1-42) would form aggregates of amyloid fibrils on the neuron plasma membranes, thus perturbing neuronal functionality. Conflicting data are available on the influence of bilayer order on Aβ(1-42) binding to membranes. In the present study, a biophysical approach was used in which isothermal calorimetry and surface pressure measurements were applied to explore the interaction of Aβ(1-42) in either monomeric, oligomeric, or fibrillar form with model membranes (bilayers or monolayers) in the liquid-ordered state that were either electrically neutral or negatively charged. In the latter case, this contained phosphatidic acid, cardiolipin, or ganglioside. The calorimetric studies showed that Aβ(1-42) fibrils, oligomers, and monomers could bind and/or be inserted into bilayers, irrespective of electric charge, in the liquid-ordered state, except that monomers could not interact with electrically neutral bilayers. The monolayer studies in the Langmuir balance demonstrated that Aβ(1-42) aggregation hindered peptide insertion into the monolayer, hindered insertion in the decreasing order of monomer > oligomer > fibril, and that lipid composition did not cause large differences in insertion, apart from a slight facilitation of monomer and oligomer insertion by gangliosides.

## 1. Introduction

Alzheimer’s disease (AD) has been identified as the major cause of late-age dementia [1]. Glenner and Wong [2] proposed that AD disease could be due to the local accumulation of the amyloidogenic protein Aβ. The so-called “amyloid (or Aβ) hypothesis” is currently considered the most potent model of AD pathogenesis, and it has generated a plethora of experimental and clinical work (see review by Selkoe and Hardy [3]). Aβ arises from the proteolysis, by β- and γ-secretases, of an amyloid precursor protein (APP). Under certain, still poorly characterized conditions, Aβ would form aggregates of amyloid fibrils deposited on the surface of neurons in dense formations known as plaques.

The processing of amyloid precursor protein (APP) into Aβ is dependent on the location of APP in the membrane, and it is very sensitive to membrane physical state and lipid composition (see reviews by Zarrouk et al. [4] and Campos-Peña et al. [5]). However, in recent studies, when a pure Aβ(1-40) peptide was mixed with monolayers of dipalmitoyl phosphatidylcholine (DPPC), which is known to undergo a temperature- and lateral pressure-dependent liquid-expanded-to-liquid-condensed bidimensional phase transition, the fibril-like structure of Aβ(1-40) appeared specifically in the liquid-expanded region [6]. Krasnobaev et al. [7] used atomic force microscopy (AFM) to study the interaction of Aβ(1-55) with membrane bilayers containing liquid-ordered (L_o_) and liquid-disordered (L_d_) lipid domains. Most of the peptide was found either in the L_d_ phase or at the boundary between ordered and disordered phases, in agreement with the data from Alvarez et al. [6]. Several studies pointed out the facilitating role of GM1 ganglioside in Aβ oligomerization [8,9,10,11]. Cholesterol was also found to positively modulate Aβ oligomerization [7,12]. Oxysterols were proposed as the link between brain cholesterol metabolism and Alzheimer’s disease [13]. Iriondo et al. [14] provided clinical evidence supporting the role of 7-ketocholesterol on axonal integrity and the involvement of cholesterol metabolism in the Aβ(1-42) generation process.

Previous studies from our laboratory demonstrated, using a combination of physical and computational techniques, that liquid-disordered bilayers consistently allowed a higher Aβ(1-42) binding than liquid-ordered ones and that low proportions (2.5–5 mol%) of negatively charged phospholipids increased the interaction [15]. More recently, Ahyayauch et al. [16] studied 1-palmitoyl-2-oleoyl phosphatidyl choline (POPC) bilayers, which exist in the fluid, or L_d_ state at room temperature, mimicking the fluidity of cell membranes, and Aβ(1-42) monomers. On the basis of molecular dynamics and Langmuir balance measurements, they showed that the peptide adsorbed onto the bilayer surface but did not become inserted into it at surface pressures compatible with the cell membrane conditions. In a separate series of studies, the binding of Aβ(1-42) peptide monomers to sphingomyelin/cholesterol (1:1 mol ratio) bilayers was studied. These bilayers are known to form stable liquid-ordered assemblies [15]. When equimolar sphingomyelin/cholesterol bilayers containing 5 mol% gangliosides were assayed by density gradient ultracentrifugation, gangliosides were seen to cause a two-fold increase in the amount of peptide bound to sphingomyelin/cholesterol vesicles and to enhance the conformational changes leading to sheet formation and, presumably, Aβ(1-42) cluster formation [17]. The sphingomyelin/cholesterol/ganglioside system was further used in a comparative study of the binding of Aβ(1-42) peptide in monomer, oligomer, or fibril forms [18]. Isothermal calorimetry (ITC) revealed that the Gibbs free energy of binding (ΔG) was virtually invariant with the aggregation state of the peptide. Measurements of monolayer surface pressure demonstrated the capacity of all peptide preparations to become inserted in lipid monolayers of any composition, although fibrils were less capable of doing so than oligomers or monomers.

The present contribution is intended to expand our understanding of Aβ42–membrane interactions using a variety of lipid compositions as well as the peptide in monomeric, oligomeric, and fibrillar forms. The thermodynamics of Aβ42 interactions with lipid vesicles were assessed with isothermal calorimetry. Moreover, lipid–peptide monolayers extended at an air–water interface were examined in a Langmuir balance to assess peptide-dependent changes in lateral pressure, indicative of peptide insertion into the monolayer. Our results underline the complexity of Aβ(1-42)–membrane interactions and the usefulness of thermodynamic equilibrium measurements in their analysis.

## 2. Results

The experiments described in this paper were performed with bilayers or monolayers, consisting essentially of sphingomyelin (SM) and cholesterol (Ch) at a 1:1 mol ratio to which small proportions of negatively charged lipids, usually 5 mol%, were added when appropriate. 1,2-Dimyristoyl phosphatidic acid (DMPA), cardiolipin, or gangliosides were included in these mixtures. Note that gangliosides bear a net negative charge due to their sialic acid components. These bilayers were shown to be in the L_o_ state under our experimental conditions [15].

### 2.1. Calorimetric Studies

**Figure 1:**
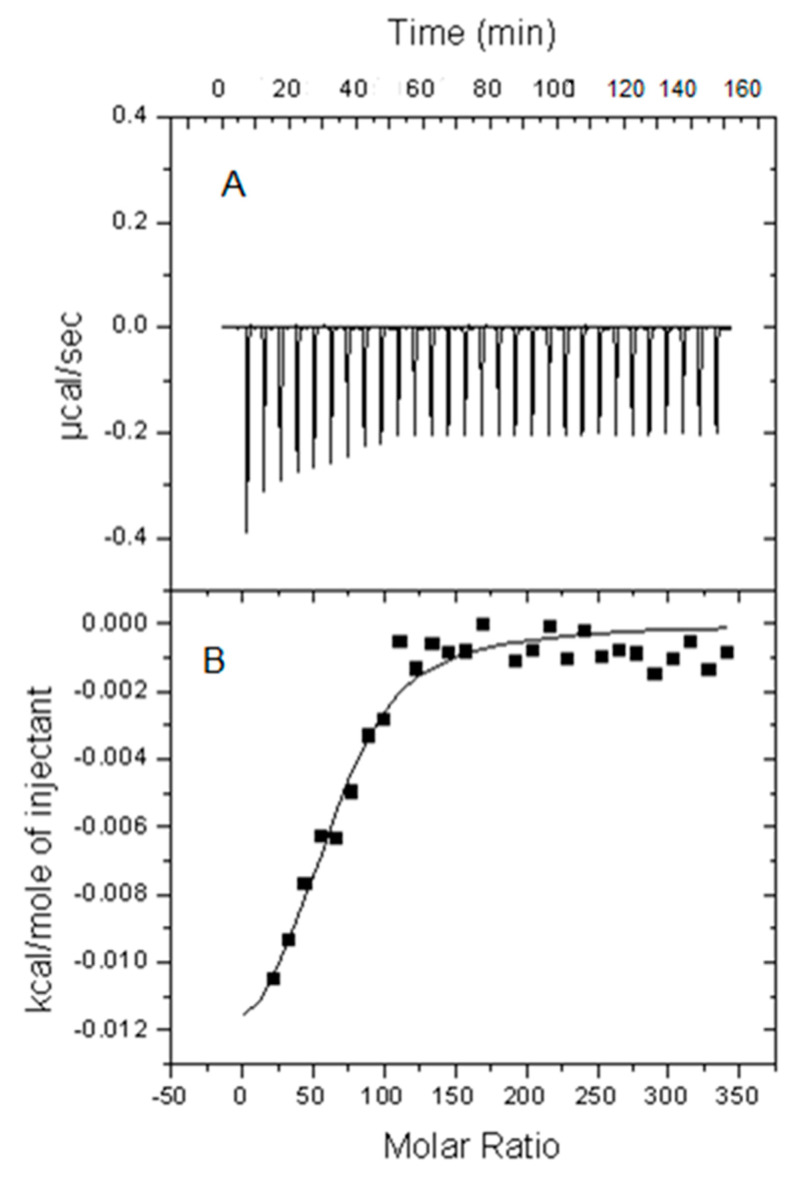
ITC calorimetric studies. (**A**) A representative titration calorimetry curve of unilamellar vesicles composed of SM/Ch (50/50 mol ratio) with Aβ(1-42) peptide fibrils, as a function of lipid/peptide mol ratio. The calorimetric trace was recorded upon successive injections of lipid vesicles into an Aβ(1-42) solution contained in the reaction cell. (**B**) Cumulative heats of the reaction, obtained from the integration of the peaks displayed in the top plot. The solid line represents the fitting of the experimental data to a partitioning model [15]. The calorimetric cell was filled with a 28 μM Aβ(1-42) solution. Lipid vesicles at 35 mM lipid concentration were injected into the cell (1.43 mL) in 10 μL steps, i.e., leading to a 143-fold dilution of lipid vesicles. Average values ± SEM (n = 3). The titration experiments were performed at 37 °C.

The interaction of Aβ(1-42) peptide in monomer, oligomer, or fibril forms with sphingomyelin/cholesterol-based bilayers was first characterized by isothermal titration calorimetry (ITC). The lipid bilayers were in the form of large unilamellar vesicles (LUV). Small amounts of LUV suspension were gradually added to a solution of Aβ peptide in the form of either monomers, oligomers, or fibrils. The measurement of heat exchanges at varying lipid/peptide ratios allowed the calculation of K_d_, K_a_, ΔH, ΔS, and ΔG of the process. A typical experiment is shown in Figure 1. The resulting thermodynamic parameters of binding, given per mol of peptide monomer, are summarized in Table 1, Table 2 and Table 3, respectively, for Aβ(1-42) peptide in either monomer, oligomer, or fibril forms. Some of the results were taken from previous publications [15,18], as indicated in the Tables, and are included here for essential comparative purposes.

With LUVs composed of SM and Ch only, in the absence of negatively charged lipids, no measurable heats of interaction were observed with Aβ(1-42) monomers (Table 1), as previously described [15,18]. However, these monomers were seen to interact with bilayers in which DMPA, total porcine brain gangliosides, or GM1 ovine brain ganglioside were incorporated (Table 1). The lipid–peptide ΔG of binding measured under our conditions was rather constant, of the order of −5 to −7 kcal/mol. This was the consequence of mutually compensating entropic and enthalpic contributions; for instance, when 5 mol% total gangliosides were present, the process was highly exothermic, suggesting the formation of multiple bonds between the peptide and (presumably) the complex sugar network of the di- and trisialogangliosides, abundant in the total brain extract. However, the binding was accompanied by a large decrease in entropy (perhaps due to the ordering of the sugar moieties), and this compensated for the more negative ΔH. ΔG corresponding to the CL-containing bilayers was unexpectedly large and negative. It could be associated with the rather low ΔS, in turn, attributable to the highly disordered bilayer containing CL linoleyl acyl chains, so the process was enthalpically drawn. Moreover, as noted previously [15], the smallest (less negative) ΔG, corresponding to the less spontaneous binding process, corresponded to the mixture containing 20 mol% DMPA. Higher doses of the negatively charged lipid had a smaller effect on binding, as predicted by molecular dynamics calculations [15], which attributed the lower binding to overall repelling electrostatic interactions: only Lys-28 appeared to have a positive interaction with the anionic lipids [19].

Studies with Aβ(1-42) in the form of soluble oligomers have the additional interest that oligomers appear to be most active from the pathogenic point of view [20]. One major difference with monomers is that oligomers were able to interact with SM/Ch bilayers even in the absence of added negatively charged lipids (Table 2). They did so with a rather robust ΔG = −7.88 kcal/mol, in which an important entropic component (T · ΔS = −5.77 kcal/mol) occurred. Mixtures containing negatively charged lipids (Table 2) bound Aβ42 oligomers with ΔG rather similar to the case of the monomers, in the 5 to 7 kcal/mol range. ΔH and ΔS values were also within relatively narrow ranges, −1 to −4 kcal/mol and 10 to 18 cal/mol, respectively (with the exception of ΔS = 1.11 cal/mol for the CL-containing mixture, a small entropy increase for a sample already quite disordered from the start).

Notable differences between monomers and oligomers (Table 1 and Table 2) are (i) the above-mentioned oligomer capacity to bind SM/Ch (1/1) bilayers, not shared by monomers; (ii) the positive values of ΔS for all mixtures involving oligomers, which happened only with the mixture containing 20 mol% DMPA and monomers; and (iii) the smaller ΔG (in absolute value) of the CL-containing mixtures with oligomers, as compared with those involving monomers. In general, the thermodynamic parameters describing binding equilibria appeared to be less dependent on bilayer composition for oligomers than for monomers. The remarkable positive values of ΔS observed with oligomers irrespective of lipid composition appear to speak in favor of a large disordering effect imposed by the oligomeric structures.

The interaction of Aβ(1-42) fibrils with LUV bilayers was assessed in the same way; the results are summarized in Table 3. Both the association/dissociation constants (K_a_/K_d_), related to the standard variation of the Gibbs’ free energy (ΔGº) and the actual changes in ΔG under our experimental conditions, were remarkably independent of the bilayer lipid composition. As discussed above for some examples of monomer binding, the constancy of ΔG was the result of compensating ΔH and ΔS values, e.g., ΔH was one order of magnitude larger in the presence of total gangliosides than in the presence of GM1 or with binary SM/Ch bilayers. However, in the latter two cases, a large, negative entropy change compensated the increased ΔH. ΔS was largest (most positive) for fibril interaction with SM/Ch than in any other system under study; this could be interpreted considering that the binary SM/Ch bilayer exhibited the largest degree of lipid order; thus, it was more perturbed than others by the fibril insertion. Conversely, in the samples containing the total ganglioside mixture, which is rich in trisialic gangliosides, the insertion of fibrils would cause a marked reorganization of the water molecules solvating the ganglioside sugar moieties, with the consequence of a decrease in entropy, compensating a large, exothermic (ΔH < 0) enthalpy change (Table 3). Note that the values in Table 1, Table 2 and Table 3 were normalized per mol of Aβ(1-42) monomer. However, the molecularity of the complex in oligomers or fibrils will be different from the monomer state (which is assumed to be 1), so the exact values of the thermodynamic parameters could diverge.

Thus, the conclusions from the calorimetric results in Table 1, Table 2 and Table 3 can be summarized as follows: (i) the binding of Aβ(1-42) fibrils, oligomers, and monomers was spontaneous (ΔG < 0) for all six lipid bilayer compositions tested, except that monomers could not interact with SM/Ch binary bilayers; (ii) Aβ(1-42) fibrils, oligomers, and monomers could bind and/or be inserted into bilayers in the liquid-ordered state, with a said exception for monomers and SM/Ch bilayers; (iii) both ΔH and ΔS were very sensitive to lipid composition, even if, in most cases, the composition was changed by only 5 mol%, and (iv) very similar values of ΔG were often attained through marked compensatory changes of ΔH and ΔS.

### 2.2. Monolayer Lateral Pressure Studies

**Figure 2:**
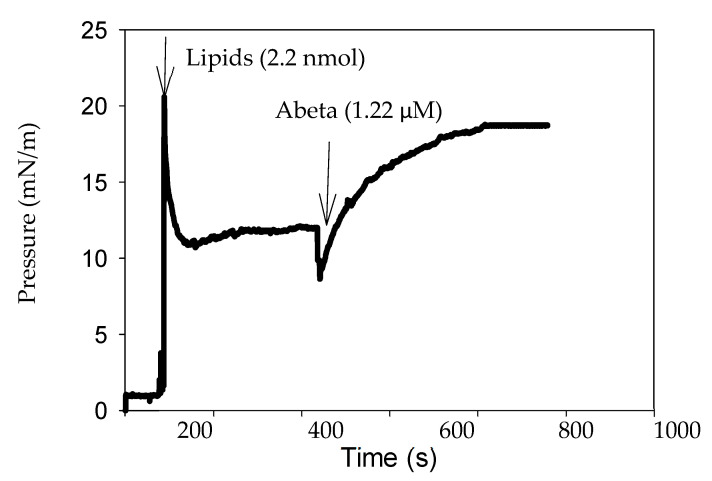
Langmuir balance studies of Aβ(1-42) interaction with membrane lipids. A representative time course of the change in surface pressure of an SM/Ch (1:1) lipid monolayer at the air–water interface upon addition of Aβ(1-42) monomers into the subphase. Aβ(1-42) stock solution was 50 μM. Aβ(1-42) final concentration in the trough was 1.22 μM. T = 22 °C.

Calorimetric studies were complemented with parallel measurements of changes in surface pressure at the air–water interface, carried out with a Langmuir balance [21,22]. In the absence of a lipid monolayer, i.e., at a ‘clean’ air–water interface, Aβ(1-42) monomers increased the surface pressure, as previously published (Figure S4 in [15]). However, neither oligomers nor fibrils did so in the absence of lipids. In a different series of experiments, an oriented lipid monolayer of the desired composition was established at the interface, then Aβ(1-42) fibrils, oligomers, or monomers were injected into the aqueous subphase, and peptide insertion into the lipid monolayer was assessed as an increase in surface pressure. A representative experiment is shown in Figure 2, in which lipids (in organic solvent) were first added on top of the water surface, and when equilibrium was reached at π_i_ ≈ 12 mN/m, by which time the solvent evaporated, the peptide was injected into the subphase. The surface pressure then increased until a new equilibrium was reached at about 800 s. Other representative time-course plots can be seen in Supplementary Figure S1.

**Figure 3:**
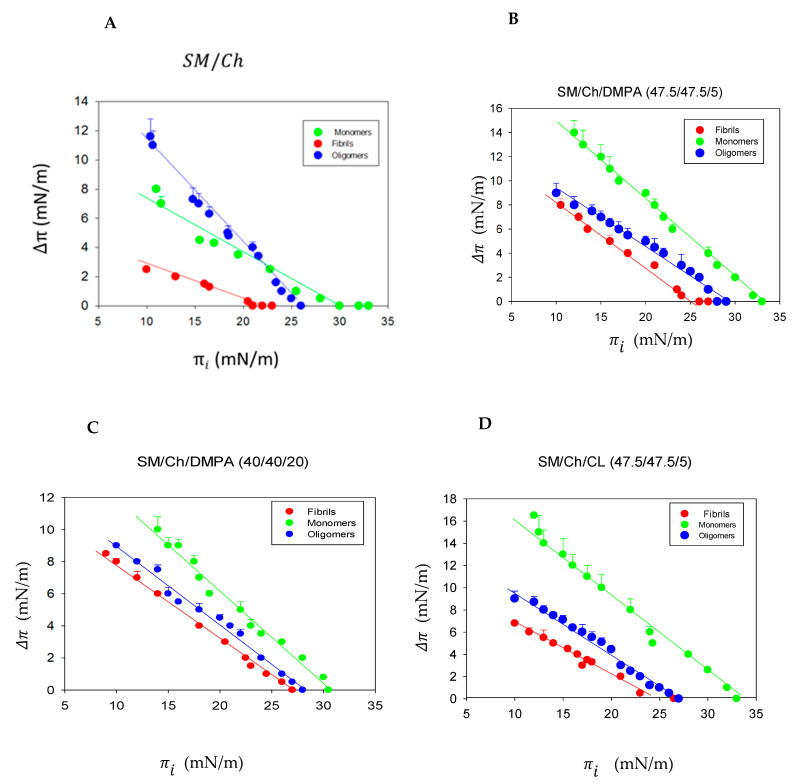
Changes in surface pressure of lipid monolayers upon insertion of Aβ(1-42) monomers, oligomers, or fibrils at varying initial pressures. (**A**) SM/Ch (1/1). (**B**) SM/Ch/DMPA (47.5/47.5/5). (**C**) SM/Ch/DMPA (40/40/20). (**D**) SM/Ch/CL (47.5/47.5/5). Average values ± S.E.M. (n = 3). Sometimes, the error bars are the same size or smaller than the symbols.

Monolayers with the same six lipid compositions tested in the ITC experiments were subjected to interaction with Aβ(1-42) fibrils, oligomers, or monomers. Measurements, as shown in Figure 1 and Figure S1, were carried out at different initial surface pressures π_i_. The results are summarized in Table 4. Details of the experiments with SM/Ch (1:1), SM/Ch/DMPA (47.5/47.5/5), SM/Ch/DMPA (40/40/20), or SM/Ch/CL (47.5/47.5/5) are shown in Figure 3 as a function of the initial surface pressure π_i_. The peptide insertion-dependent increase in surface pressure Δπ decreased with increasing initial pressures π_i_ until Δπ = 0 at the limit initial pressure, beyond which no insertion could occur. The limit π_i_ or maximal insertion pressure decreased for all lipid mixtures in the order of monomer > oligomer > fibril (Figure 3 and Table 4), reasonably suggesting that the size of the peptidic product to be inserted in the monolayer imposed certain restrictions. It should be noted in this respect that the surface pressure of cell membranes was estimated, albeit with large maximum and minimum fluctuations, at an average π ≈ 30 mN/m [23]. Thus, the data in Table 4 would suggest that the Aβ(1-42) monomers and oligomers, in the presence of gangliosides, would be able to insert into the cell membranes but not the Aβ(1-42) fibrils nor oligomers in the absence of gangliosides. However, the translation of monolayer data to cell membranes should be performed with precaution.

Table 4 also summarizes the Δπ values caused by peptide insertion into monolayers that existed initially at π_i_ = 16 mN/m. The latter figure was chosen arbitrarily in a π_i_ region in which sizable Δπ values occur. Δπ at π_i_ = 16 mN/m provided a semi-quantitative estimation of the affinity of an Aβ42 sample for the lipid monolayer under conditions where insertion is possible and easy. In general, Δπ at π_i_ = 16 mN/m decreased in the order monomer > oligomer > fibril (Figure 3 and Table 4), i.e., in the same order as the limit π_i_, thus both parameters reinforced each other mutually. An exception occurred for the monomer insertion into SM/Ch monolayers, whose limit π_i_ was larger than that of oligomers or fibrils, even if, at π_i_ = 16 mN/m (indeed at any π_i_ < 22 mN/m), Δπ was smaller than that of oligomers (Figure 3A and Table 4). In the absence of negative charges in the monolayer, these results may reflect the almost pure hydrophobic peptide binding, at variance with the situation with added negative lipids. Then, the Aβ oligomers, suspected to be the most pathogenic form [20], would also be the most hydrophobic one.

**Figure 4:**
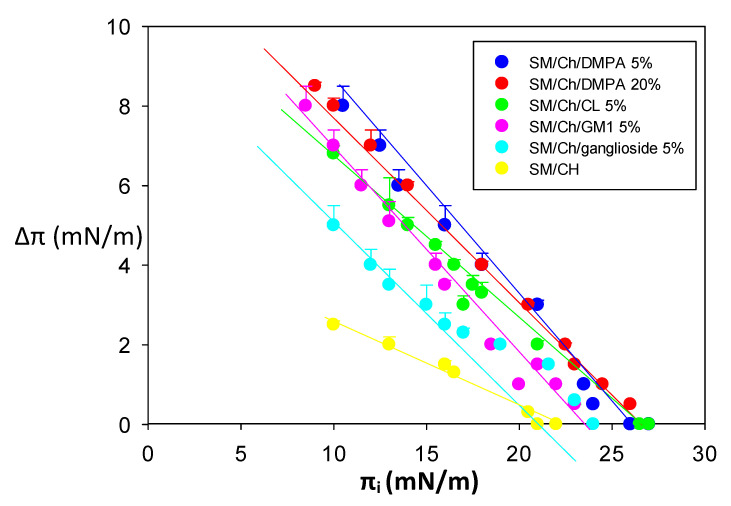
Langmuir balance studies of Aβ(1-42) fibril interaction with membrane lipids at varying initial pressures. Code color for monolayer lipid compositions: see inset. Average values ± S.E.M. (n = 3). Sometimes, the error bars are the same size or smaller than the symbols.

A complementary view of our monolayer studies is given in Figure 4, in which the interactions of all six lipid monolayers with Aβ(1-42) fibers are shown together. In agreement with the data in Table 4, SM/Ch departed from the behavior of the remaining monolayers in that the slope of the Δπ vs. π_i_ plot was smaller, and Δπ at π_i_ = 16 mN/m as well as the limit π_i_ were lower. In general, SM/Ch monolayers appeared to be scarcely accessible to the Aβ(1-42) fibrils, in any case, less so than the other lipid compositions.

In conclusion, the monolayer studies at the air–water interface demonstrate that (i) Aβ(1-42) aggregation hindered peptide insertion into the monolayer, hindered insertion in the decreasing order of monomer > oligomer > fibril; (ii) lipid composition did not cause large differences in insertion, apart from slight facilitation of monomer and oligomer insertion by gangliosides; and (iii) SM/Ch constituted an exception to the above rule in that it exhibited a particularly low binding to fibrils.

## 3. Discussion

The above results make pertinent a discussion on (i) a comparison of the calorimetric and surface pressure data, (ii) the influence of the peptide aggregation state, and (iii) the role of bilayer properties (physical state, electric charge) on the binding of Aβ(1-42) to membranes.

### 3.1. Two Techniques, One Phenomenon?

The two techniques used in this study are among the most divergent ones in membrane biophysical studies. Calorimetry uses, in our hands, vesicles surrounded by lipid bilayers, while surface pressure is studied on lipid monolayers. Vesicle bilayers are curved, while monolayers are flat. Bilayers as models are closer than monolayers to the cell membranes, while monolayers allow an almost infinite variation of lipid compositions and lateral pressures, which could better illustrate certain aspects of lipid–protein interaction and are not always accessible to the bilayer models. Detailed discussions on the virtues and limitations of each methodology can be found in the literature: Maget-Dana [21] or Radhakrishnan and McConnell [24] for surface pressures, Heerklotz and Seelig [25] or Freire et al. [26] for isothermal titration calorimetry. However, both techniques have an important characteristic in common, namely that they are both equilibrium techniques in the sense that they provide data that are in themselves, or directly translated into, thermodynamic functions of a state. Measurements in equilibrium have the immense advantage of allowing immediate reproducibility and a comparison of results. Arguments on the supposed advantages of one technique over the other would be, in our view, futile. They should rather be considered complementary to each other, shedding light on different facets of the same event.

In our case, increased surface pressures in monolayers in the presence of peptides, particularly when, as in our case, they are dose-dependent, are a clear indication of peptide/protein insertion in the monolayer, i.e., interaction with the phospholipid acyl chains [27]. Calorimetry, in turn, reports on interactions in a broader sense. Heat exchanges could arise from polar or electrostatic interactions involving the polar part of lipids, from hydrophobic bonding involving the bilayer acyl chains, or from a combination of the aforementioned. It is, thus, not surprising that results obtained from both techniques do not necessarily match. With this caveat in mind, it should be observed that binding of Aβ(1-42) fibrils, oligomers, and monomers to monolayers or bilayers of any of the compositions tested was observed with both techniques, with the exception of monomer binding to SM/Ch, which occurred with monolayers but not with bilayers. Note that Δπ at π_i_ = 16 mN/m was lower (≈1/3) for monomers in SM/Ch than for any other mixtures, suggesting a lower insertion ability (Table 3, Figure 4). Other properties, e.g., the facilitating effect of negatively charged lipids, were seen in all cases by both techniques (Table 1, Table 2, Table 3 and Table 4). In particular, the binding of fibrils was very similar in the cases of all the monolayers and bilayers, as assayed by any of the two techniques (Table 3 and Figure 4). In general, the above data support the compatibility of the isothermal calorimetry and Langmuir monolayer techniques, even if occasionally the corresponding results fail to overlap, presumably due to the intrinsic differences in the physical events that are being measured.

### 3.2. The Peptide Aggregation State

There is a relative scarcity of data concerning the influence of the peptide aggregation state and the incorporation of Aβ(1-42) into membranes. An important caveat in our study is that, even if all measurements start with Aβ(1-42) in a predominant monomer, oligomer, or fibril form, it cannot be ruled out that interconversions between these forms occur during the measurements. However, the results in this and previous papers [15,18] consistently show differences in the lipid interaction with monomers, oligomers, or fibrils. Thus, in the absence of direct proof, it can be safely assumed that a predominant form of Aβ(1-42) occurs in each kind of experiment. An additional limitation of our study would be that several simultaneous or almost simultaneous events might be taking place, obscuring the interpretation of the physical measurements. From the published evidence, at least the Aβ release from APP, Aβ binding/insertion, and Aβ aggregation can be conceptually distinguished. The experiments in this paper show how binding is not easily separated from insertion; even if the Langmuir balance measurements report on peptide insertion in the monolayer, the calorimetric measurements provide information on bilayer binding + insertion (if not also, in part, aggregation). Ahyayauch et al. [18] specifically addressed the question of the influence of the Aβ(1-42) aggregation state on ganglioside-containing bilayer binding. They found that insertion, assessed by surface pressure measurements, was more difficult for fibrils than for monomers or oligomers. However, the ΔG of binding, derived from isothermal calorimetry measurements, indicated robust spontaneous binding in all cases (ΔG ≈ −7 kcal/mol Aβ) and no clear influence of Aβ(1-42) aggregation state or type of ganglioside in the membrane. 

The data in this paper were more complex; they showed similar thermodynamic parameters for Aβ(1-42) binding/insertion in bilayers containing 5 mol% DMPA, irrespective of the peptide aggregation state, but a preference, decreasing in the order of monomer > oligomer > fibril, for insertion in monolayers. When DMPA concentration was 20 mol%, ΔG indicated a less spontaneous process for monomers (−5.3 kcal/mol) than for oligomers or fibrils (≈−7.5 kcal/mol for both) (Table 1, Table 2 and Table 3), while monomers appeared to insert more readily in the monolayers (Figure 3C). Bilayers including 5 mol% CL in their composition allowed interaction with Aβ(1-42), decreasing in the order of monomer > fibril > oligomer (Table 1, Table 2 and Table 3), while insertion in monolayers of the same composition decreased in the order of monomer > oligomer > fibril (Figure 3D). Studies allowing the independent assessment of at least peptide binding, insertion, and aggregation will be required for a proper interpretation of the above results. Meanwhile, it might be provisionally concluded that, from the biophysical data, the monomers appear to interact with lipids more readily than fibrils, with oligomers showing intermediate properties. This would be consistent with the idea that the membrane-bound monomer would act as a primer for oligomer/fibril formation. Thus, the question as to which peptide-aggregation form is more pathogenic would not have a simple answer because, even if the aggregates appear to be more functionally disturbing, they would not be so easily formed in the absence of membrane binding by monomers. Note that, among the various products of γ-secretase action on APP, only Aβ(1-42) was selected for this study because of its perceived higher toxicity [28]. It would be interesting to compare, in parallel studies, the bilayer binding and cell toxicity of the various Aβ peptides.

### 3.3. The Bilayer Physical Properties

The extent to which liquid-ordered and liquid-disordered (L_d_) domains coexist in cell membranes, or even the mere existence of liquid-ordered (L_o_) domains in cells, is still an object of debate [29,30,31,32,33]. For the specific case of Aβ–membrane interactions, Krasnobaev et al. [7] observed, using atomic force microscopy, that transmembrane fragment APP672-726 (corresponding to Aβ1-55) is located either in the L_d_ phase or at the boundary between ordered and disordered phases but not in L_o_ domains. We already observed that L_d_ bilayers consistently allowed a higher Aβ(1-42) binding than L_o_ ones [15]. This would cast doubts on the interest of studying Aβ interactions with L_o_ membranes. However, the situation may be more complex. Conflicting data are available on the influence of bilayer order on Aβ binding to membranes. Bilayer lipid order has usually been modulated by changing cholesterol levels in the bilayers. Results suggesting increased Aβ deposition on Ch-rich (ordered) domains have been published [34,35], while other authors have observed increased peptide aggregation under the opposite conditions, i.e., in cell membranes when Ch synthesis was inhibited or from which Ch was removed [35,36]. Experiments in cell membranes have the problem that two concurring phenomena are taking place in membranes, namely APP hydrolysis and Aβ deposition/aggregation. The former appears to occur preferentially in more ordered domains so that Aβ would be generated in those domains, while deposition/aggregation could well take place, partly or totally, in different membrane regions, e.g., adjacent disordered domains. Note, however, that Siniscalco et al. [37] support the idea that the nascent Aβ polypeptides are immediately bound to the underlying bilayer, in principle, an ordered lipid structure. In any case, there is no agreement on the physical properties of the domains where newly released Aβ binds membranes in cells. The case is further complicated when one considers that there is a gradual, continuous gradient of lipid order between the various kinds of membrane domains. Canonical L_o_ domains [29,38] are formed by Ch and fully saturated phosphatidylcholines, or sphingomyelins, none of which are major phospholipid components in cell membranes; thus, more or fewer ordered domains may be found in cells rather than the canonical (almost ideal) L_d_/L_o_ paradigm. In these circumstances, it is difficult to dismiss experiments carried out under any specific conditions, L_d_ or L_o_, as long as they are carefully characterized and monitored. In our case, it was checked that, even in the presence of 5 mol% or 20 mol% DMPA [15] or of 5 mol% gangliosides (unpublished data), bilayers based on an equimolar SM/Chol composition remain in a L_o_ phase.

The putative influence of bilayer lipid order on Aβ(1-42) binding would probably occur through hydrophobic interactions, but electrostatic interactions cannot be neglected, particularly when abundant experimental proof of lipid net charge effects on Aβ(1-42) binding is available. In a previous study [15], it was found that negatively charged lipids helped in binding Aβ(1-42) monomers to the bilayer. This was in agreement with other authors’ results [39,40]. The above results (Table 1, Figure 4) show as well that negative charges in the bilayers enhance Aβ(1-42) binding, particularly, but not only, in monomer form. The positively charged Lys-28 residue is a good candidate to initiate Aβ(1-42) binding to negatively charged bilayers [15]. Robinson et al. [41], using atomic force microscopy on dioleoyl phosphatidylcholine supported lipid bilayers, found that the addition of 10 mol% dioleoyl phosphatidylserine (negatively charged at neutral pH) increased Aβ(1-42) binding and oligomerization. Ahyayauch et al. [18] showed that GM1 ganglioside was a major enhancer of Aβ binding to lipid bilayers in the L_o_ state. In fact, gangliosides have repeatedly been described as promoting oligomer and fibril formation, in which their net negative charge is presumably involved [8,9,10,11,42]. The ganglioside effect may be non-linear with the dose. Alvarez et al. [43] described that, above a certain concentration, the fibrils dissolve into irregular domains and then disappear, thus adding another dimension to the complexity of the system.

### 3.4. From Model to Cell Membranes

This investigation has been carried out on simplified membrane models. As stated above, the translation of monolayer or bilayer data to cell membranes should be taken with precaution. Some considerations are relevant.

Concerning amyloidogenic processing, it begins with APP cleavage by β-secretase (BACE) in the plasma membrane, generating a C-terminal fragment (C99) and releasing soluble APP β (sAPPβ) into the extracellular space. C99 is then cleaved by the γ-secretase enzyme complex generating amyloid-β protein precursor intracellular domain (AICD) and Aβ. Aβ peptides are released into the extracellular space. As a result, Aβ peptides varying from 30 to 43 amino acids in length are secreted into the extracellular space, where they constitute the seed for the formation of Aβ-amyloid aggregates, a key step in the formation of amyloid plaques [44,45].

With respect to the anionic lipids studied in the present investigation, gangliosides are localized in the outer leaflet of the plasma membrane. Phosphatidic acid is usually found in the opposite inner face of the plasma membrane. Cardiolipin is, in turn, a lipid specific to mitochondria [46,47].

In the abovementioned view, studying Aβ(1-42) interaction with model membranes containing these lipids can be useful in modeling different cellular situations: (*i*) ganglioside-containing mono- and bilayers are directly related to the process of Aβ extracellular aggregation, formation of Aβ-amyloid extracellular aggregates, and plasma membrane-mediated cellular toxicity of Aβ. (*ii*) Cardiolipin is particularly associated with Aβ–mitochondria interaction, and it is worth reiterating that mitochondrial impairment is a characteristic of AD [47]. Thus, model membranes containing this lipid could be utilized to investigate the possible toxicity of Aβ to these organelles. (*iii*) Concerning PA, this lipid is endocytosed together with APP and Aβ-related enzymes [48]. The endocytic generation of Aβ is progressing with age [49]. Thus, membrane models containing phosphatidic acid could be used to investigate the interactions of intracellular Aβ. In summary, taking the necessary precautions when translating the model membrane data to the living cell, the results in this paper can be relevant to various aspects of AD pathophysiology at the molecular and cell levels. 

## 4. Materials and Methods

### 4.1. Materials

Aβ(1-42) (purity > 90%) was generously supplied by Mario Negri Institute (Milan, Italy). Gangliosides (ammonium salts) were also from Avanti (Alabaster, AL, USA): GM1 (ovine brain, 860065), and a total ganglioside extract from porcine brain (860053) containing mainly GM3, GM2, GM1, Fuc-GM1, GD1a, GD1b, Fuc-GD1b, GT1b, and GQ1b [50]. Further details about Materials can be found in [15,16,17,18].

### 4.2. Aβ(1-42) Sample Preparation

Aβ(1-42) samples were prepared as described by Gobbi et al. [51]. See [15,18] for other details.

### 4.3. Monomers

The peptide film was resuspended immediately prior to use in Tris Buffer (10 mM Tris, 150 mM NaCl, 1 mM EDTA pH 7.4). The monomeric form was checked by IR (absence of the 1667 cm^−1^ signal) [17] and thioflavin fluorescence (constancy of fluorescence emission after 6 h) [17,52]. ITC experiments took 5 h on average; Langmuir balance measurements were completed in <1 h.

### 4.4. Oligomers

To obtain Aβ(1-42) oligomeric forms, the monomeric peptide solutions were diluted to 100 μM in 10 mM Tris, 150 mM NaCl, 1 mM EDTA, pH 7.4 buffer, and incubated for 24 h at 4 °C. The presence of oligomers in these preparations was confirmed by IR (presence of the 1667 cm^−1^ signal) [17] and increased thioflavin T fluorescence emission [16,17,52].

### 4.5. Fibrils

To obtain Aβ(1-42) fibrils, the monomeric peptide solutions were diluted to 100 μM in buffer acidified to pH 2.0 with HCl and left for 48 h at 37 °C, following the procedure by Dahlgren et al. [53], as described by Gregori et al. [54]. The predominant presence of fibrils in those preparations was confirmed by the abovementioned authors using atomic force microscopy and dynamic light scattering. The almost exclusive β-structure of the fibrils was maintained for at least 6 h after they were transferred to pH 7.4 buffer, according to IR and Thioflavin T fluorescence measurements.

### 4.6. Liposome Preparation

Large unilamellar vesicles (LUVs) were prepared by the extrusion method, using polycarbonate filters with a pore size of 0.1 μm (Nuclepore, Pleasanton, CA, USA). See details in [16,17,18].

### 4.7. Isothermal Titration Calorimetry

The enthalpy change upon partitioning of monomeric Aβ(1-42) into SM/Ch/ganglioside LUVs could be measured with high-sensitivity ITC. ITC was performed using a model VP-ITC high-sensitivity titration calorimeter (MicroCal, Northampton, MA, USA). See details in [15,16,17,18]. The obtained isotherm was used to determine the thermodynamic parameters of partitioning [15,55]. Thioflavin T fluorescence and IR spectra showed that the fibril structure was maintained at pH 7.4 beyond the typical 5 h duration of the ITC experiment.

The experimental data were analyzed using the Origin 6.0 software as provided by Microcal. For the fitting of the data to the partitioning model, the PartiRel program, developed by Heerklotz et al. [25,56], was used with permission of the authors.

### 4.8. Lipid Monolayer Measurements

Monolayers at the air–water interface in a Langmuir balance were studied at 22 °C [15,18,57]. Lateral pressure experiments were carried out with a MicroTrough S system from Kibron (Helsinki, Finland) under constant stirring. 

## 5. Concluding Remarks

(a) Interaction of the amyloidogenic Aβ(1-42) peptide with cell membranes can be mimicked using model lipid monolayers or bilayers. Interaction with bilayer membranes can adopt at least two different forms: adsorption onto the membrane surface or insertion into it. The expression ‘membrane binding’ is often used to encompass both situations. Adsorption and insertion should not be considered as two different end-points of a process: adsorption can be a reversible, intermediate step leading to either insertion or desorption, while insertion is usually an irreversible event. Of the two main techniques used in the present study to measure lipid–protein interaction, increased surface pressures, as detected in the Langmuir balance, are usually interpreted in terms of peptide insertion into the monolayer. However, insertion into a monolayer is not equivalent to insertion in a bilayer; the former can occur without the latter. The calorimetric assessment of the interaction does not allow, in turn, to distinguish between adsorption and insertion; thus, our observations are globally referred to as peptide binding.
(b) It is generally accepted that Aβ monomers associate among themselves, ultimately giving rise to micrometer-sized amyloid plaques, monomers giving rise to oligomers, then to fibrils. These early aggregation steps can occur in aqueous media, although they might be facilitated/catalyzed by a primer consisting of a membrane-bound peptide molecule. Some of the above experimental results appear to indicate that preparations highly enriched in either monomers, oligomers, or fibrils interact differentially with membranes; this does not exclude that multiple equilibria (monomers, oligomers, fibrils) are simultaneously occurring. Thus, any quantitative analysis of Aβ amyloid formation in membranes must take into account these complex inter-peptide and peptide–lipid interactions.
(c) Model membrane bilayers can be prepared, among others, in the liquid-disordered and the liquid-ordered states that could be respectively represented, e.g., by the lamellar phases of egg phosphatidylcholine and of an equimolar sphingomyelin/cholesterol mixture. However, these are extreme examples that may or may not correlate with cell membranes. A realistic interpretation of results obtained with those kinds of compositions should keep in mind that natural membranes are not laterally homogeneous so that domains with different degrees of molecular order can coexist. Moreover, within a given domain, a gradual spectrum of molecular order may occur between the fully ordered and the fully disordered states. Peptides and proteins tend to insert more easily into more disordered domains/membranes or at the interfaces between ordered and disordered domains.
(d) The above data with monolayers and bilayers based on equimolar sphingomyelin/cholesterol mixtures correspond to a hypothetical cell membrane situation in which Aβ(1-42) binding is very difficult. They would represent a basal or minimal binding that would increase in the presence of negatively charged lipids (at concentrations compatible with the liquid-ordered state), particularly for monomers.
(e) A reasonable hypothetical scenario would contemplate that, even in highly ordered domains, Aβ(1-42) monomers would be able to bind in the presence of some negatively charged lipids, in turn interacting with basic amino acid residues in Aβ(1-42), e.g., Lys-28. The membrane-bound monomer would then act as a catalyst (or a primer) for β-sheet formation, oligomerization, fibril formation, and ultimately, plaque deposit.

## Figures and Tables

**Table 1:** Thermodynamic parameters for the interaction of Aβ(1-42) monomers with unilamellar vesicles. Average values ± SEM (n = 3).

Monomer	SM/Ch/DMPA (47.5/47.5/5) ^a^	SM/Ch/DMPA (40/40/20) ^a^	SM/Ch/CL (47.5/47.5/5) ^a^	SM/Ch/GM1 (47.5/47.5/5) ^b^	SM/Ch/T. Gang. (47.5/47.5/5) ^b^	SM/Ch (1/1)
K_a_ (M^−1^) (×10^4^)	3.09 ± 0.9	58.2 ± 8.0	16.0 ± 2	9.4 ± 0.3	28.0 ± 2	-
K_d_ (μM)	32 ± 1.1	1.71 ± 12.5	6.25 ± 1.2	10.6 ± 0.3	3.5 ± 0.2	-
∆H (kcal/mol)	−7.3 ± 0.05	−2.8 ± 0.19	−12.6 ± 1.4	−15.6 ± 1.4	−108.2 ± 12	-
∆S (cal/mol K)	−3.0 ± 0.1	8.1 ± 0.2	−1.68 ± 0.4	−27.4 ± 1.8	−324 ± 8	-
∆G (kcal/mol)	−6.4 ± 0.1	−5.3 ± 0.01	−11.96 ± 0.6	−7.1 ± 0.6	−7.8 ± 0.6	-

^a^ Ahyayauch et al. [15]; ^b^ Ahyayauch et al. [18].

**Table 2:** Thermodynamic parameters for the interaction of Aβ(1-42) oligomers with unilamellar vesicles. Average values ± SEM (n = 3). Note that values were computed per mol of monomer.

Oligomer	SM/Ch/DMPA (47.5/47.5/5)	SM/Ch/DMPA (40/40/20)	SM/Ch/CL (47.5/47.5/5)	SM/Ch/GM1 (47.5/47.5/5) ^a^	SM/Ch/T. Gang. (47.5/47.5/5) ^a^	SM/Ch (1/1) ^a^
K_a_ (M^−1^) (×10^4^)	1.22 ± 0.23	16.4 ± 1.3	51 ± 1	11 ± 0.1	21.6 ± 0.8	36.7 ± 0.7
K_d_ (µM)	81.9 ± 4.3	6.1 ± 0.77	2.0 ± 9.09	9.1 ± 0.91	4.6 ± 0.1	2.72 ± 0.01
∆H (kcal/mol)	−1.19 ± 0.15	−4.27 ± 0.53	−4.92 ± 0.43	−0.83 ± 0.02	−2.43 ± 0.05	−2.11 ± 0.01
∆S(cal/mol K)	14.9 ± 1.5	10.1 ± 1.2	1.11 ± 0.52	11.2 ± 0.5	16.6 ± 0.4	18.6 ± 0.1
∆G (kcal/mol)	−5.80 ± 0.34	−7.37 ± 0.25	−5.25 ± 0.34	−4.25 ± 0.07	−7.57 ± 0.09	−7.88 ± 0.04

^a^ Ahyayauch et al. [18].

**Table 3:** Thermodynamic parameters for the interaction of Aβ(1-42) fibrils with unilamellar vesicles. Average values ± SEM (n = 3). Note that values were computed per mol of monomer.

Fibril	SM/Ch/DMPA (47.5/47.5/5)	SM/Ch/DMPA (40/40/20)	SM/Ch/CL (47.5/47.5/5)	SM/Ch/GM1 (47.5/47.5/5) ^a^	SM/Ch/T. Gang. (47.5/47.5/5) ^a^	SM/Ch (1/1) ^a^
K_a_ (M^−1^)(×10^4^)	18.5 ± 2.4	27.6 ± 8.2	28.6 ± 0.8	18.0 ± 0.7	13.0 ± 0.3	21.0 ± 0.4
K_d_ (µM)	5.4 ± 0.18	3.6 ± 0.12	3.5 ± 0.3	5.6 ± 0.2	7.6 ± 0.3	4.8 ± 0.7
∆H (kcal/mol)	−6.14 ± 0.3	−8.41 ± 0.405	−4.31 ± 0.07	−1.78 ± 0.05	−29.6 ± 0.4	−0.87 ± 0.05
∆S (cal/mol K)	4.31 ± 0.08	−2.22 ± 0.05	11.1 ± 0.2	18.3 ± 0.7	−71.9 ± 1.8	21.6 ± 0.2
∆G (kcal/mol)	−7.47 ± 0.02	−7.72 ± 0.03	−7.75 ± 0.06	−7.45 ± 0.08	−7.27 ± 0.05	−7.56 ± 0.04

^a^ Ahyayauch et al. [18].

**Table 4:** Changes in surface pressure of lipid monolayers upon insertion of Aβ(1-42) peptide: a summary of results. Data derived from experiments as in Figure 2, Figure 3 and Figure 4. Average values ± SEM (n = 3).

Lipid Composition		∆π (mN/m) at π_i_ = 16 mN/m	Maximal Insertionpressures
SM/Ch ^a^	Monomers	4.5 ± 0.3	32 ± 0.5
	Oligomers	6.0 ± 0.2	26 ± 0.0
	Fibrils	1.94 ± 0.2	21 ± 0.4
SM/Ch/DMPA (5%)	Monomers	11.0 ± 0.6	32 ± 0.0
	Oligomers	6.5 ± 0.7	28 ± 0.2
	Fibrils	5.0 ± 0.4	26 ± 0.3
SM/Ch/DMPA (20%)	Monomers	9.2 ± 0.5	30 ± 0.1
	Oligomers	5.5 ± 0.1	27 ± 0.2
	Fibrils	5.3 ± 0.2	26 ± 0.4
SM/Ch/CL (5%)	Monomers	12.0 ± 1	32 ± 0.0
	Oligomers	6.4 ± 0.2	27 ± 0.1
	Fibrils	4.2 ± 0.1	26 ± 0.2
SM/Ch/GM1 (5%) ^a^	Monomers	17.1 ± 0.4	34 ± 0.2
	Oligomers	12.4 ± 0.5	32 ± 0.0
	Fibrils	3.5 ± 0.5	24 ± 0.5
SM/Ch/total ganglioside (5%) ^a^	Monomers	11.7 ± 0.5	34 ± 0.0
	Oligomers	8.5 ± 0.6	31 ± 0.3
	Fibrils	2.7 ± 0.2	24 ± 0.4

^a^ Ahyayauch et al. [18].

## Data Availability

The data presented in this study are available upon request from the corresponding author.

## Supplementary Materials

The following supporting information can be downloaded at: https://www.mdpi.com/article/10.3390/biom14030298/s1. Figure S1: Langmuir balance studies of Aβ42 interaction with membrane lipids. Representative time courses of the change in surface pressure of a lipid monolayer, at the air-water interface, upon addition of Aβ42 monomers into the subphase. Aβ42 stock solution was 50 μM. Aβ42 final concentration in the trough was 1.22 μM. T = 22 °C. Monolayer compositions and state of aggregation of Aβ are given on top of each plot.

## Abbreviations

Ch, cholesterol; CL, cardiolipin; DMPA, dimyristoyl phosphatidic acid; ITC, isothermal calorimetry; PA, phosphatidic acid; SM, sphingomyelin.
