# Supplementary material for: The Influence of Lipid Electric Charge on the Binding of Aβ(1–42) Amyloid Peptide to Bilayers in the Liquid-Ordered State
Source: Biomolecules. 2024 Mar 1;14(3):298. doi: 10.3390/biom14030298 (PMC10967883; doi:10.3390/biom14030298)
Supplement: Supplementary file 1 [file biomolecules-14-00298-s001.zip › biomolecules-2856740-supplementary.pdf]

SUPPLEMENTARY FIGURE

The influence of lipid electric charge on the binding of β-amyloid (1–42) peptide to bilayers in the liquid-ordered state

Ahyayauch et al.

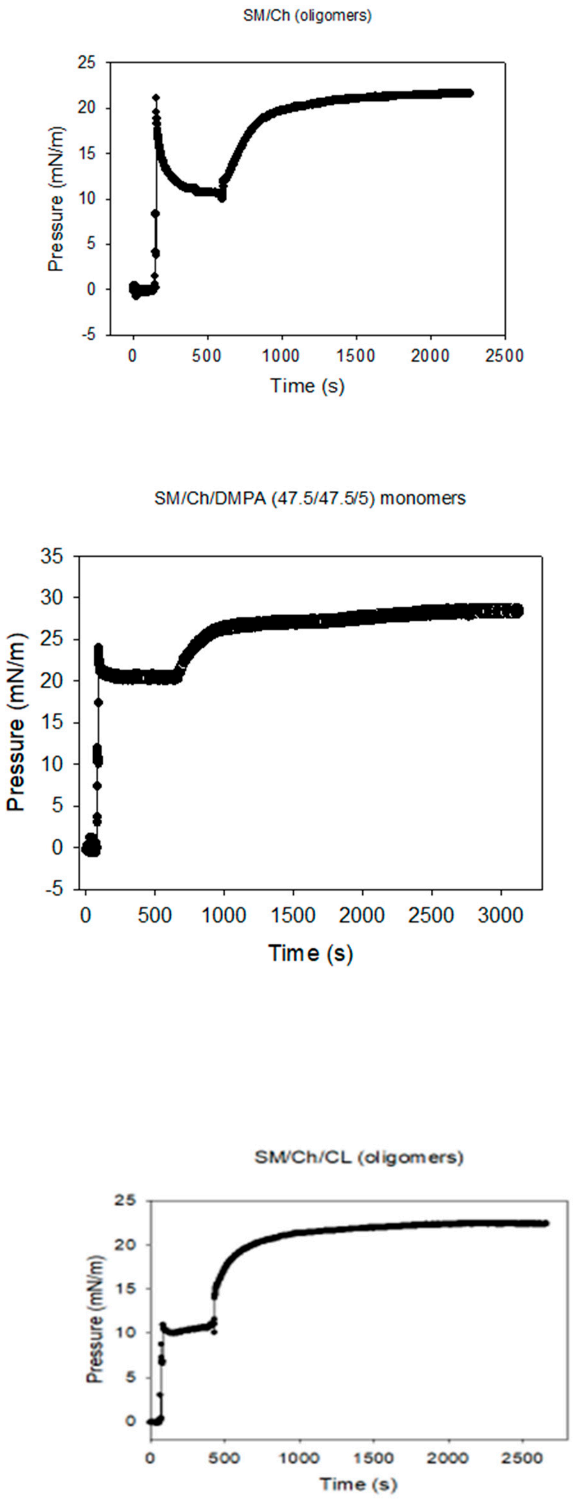

Figure S1. Langmuir balance studies of Aβ42 interaction with membrane lipids. Representative time courses of the change in surface pressure of a lipid monolayer, at the air-water interface, upon addition of Aβ42 monomers into the subphase. Aβ42 stock

solution was 50 μM. Aβ42 final concentration in the trough was 1.22 μM. T = 22 °C. Monolayer compositions and state of aggregation of Aβ are given on top of each plot.
